# Collectanea Medica: Consisting of Anecdotes, Facts, Extracts, Illustrations, &c.

**Published:** 1821-10

**Authors:** 


### COLLECTANEA MEDICA:

CONSISTING OF

### ANECDOTES, FACTS, EXTRACTS, ILLUSTRATIONS, &c.

*Relating to the History or the Art of Medicine, and the Auxiliary Sciences.*

Floriferis ut apes in saltibus omnia libant,

Omnia nos itidem depascimur aurea dicta.

### *Continuation of our Abstract of* Baron Humboldt's *Dissertation on Isothermal Lines, and the Distribution of Heat over the Globe.*

AFTER what has already been stated respecting the limits between which the annual heat divides itself on the same isothermal curve, it will be seen how far we are authorized to say, that the *coffee-tree*, the *olive*, and the *vine*, in order to be productive, require mean temperatures of 64·4, 60·8, and 53·6, Fahr. These expressions are true only of the same system of climate,—for example, of the part of the Old World which stretches to the west of the meridian of Mont Blanc; because, in a zone of small extent in longitude, while we fix the annual temperatures, we determine also the nature of the summers and the winters. It is known, likewise, that the olive, the vine, the varieties of grain, and the fruit-trees, require entirely different constitutions of the atmosphere. Among our cultivated plants, some, slightly sensible of the rigors of winter, require very warm, but not long, summers; others require summers rather long than warm; while others, again, indifferent to the temperature of summer, cannot resist the great colds of winter. Hence it follows, that, in reference to the culture of useful vegetables, we must discuss three things for each climate: the mean temperature of the entire summer,—that of the warmest month,—and that of the coldest month. I have published the numerical results of this discussion in my *Prolegomena de Distributione Geographica Plantarum, secundum Cœli Temperiem*; and I shall confine myself at present to the limits of culture of the olive and the vine. The olive is cultivated in our continent between the parallels of 36° and 40°, wherever the annual temperature is from 62·6 to 58·1, where the mean temperature of the coldest month is not below from 41·0 to 42·8, and that of the whole summer from 71·6 to 73·4. In the New World, the division of heat between the seasons is such, that, on the isothermal line of 58·1, the coldest month is 35·6, and that the thermometer sometimes sinks there even, during several days, from 14° to 10·4. The region of potable wines extends, in Europe, between the isothermal lines of 62·6 and 50°, which correspond to the latitudes of 36° and 48°. The cultivation of the vine extends, though with less advantage, even to countries whose annual temperature descends to 48·2 and to 47·48; that of winter to 33·8, and that of summer to 66·2 and 68°. These meteorological conditions are fulfilled in Europe as far as the parallel of 50°, and a little beyond it. In America, they do not exist farther north than 40°. They have begun, indeed, some years ago, to make a very good red wine to the west of Washington, beyond the first chain of mountains, in the valleys which do not extend beyond 38° 54′ of lat. On the continent of Western Europe, the winters, whose mean temperature is 32°, do not commence till on the isothermal lines of 48·2 and 50°, in from 51° to 52° of latitude; while, in America, we find them already on the isothermal lines of from 51·8 to 53·6, under from 40° to 41° of latitude.

If, instead of considering the natural inflexions of the isothermal lines,—that is to say, those that propagate themselves progressively at great intervals of longitude,—we direct our attention to their partial inflexions, or to particular systems of climates occupying a small extent of country, we shall still find the same variations in the division of the annual heat between the different seasons. These partial inflexions are most remarkable:

1st. In the Crimea, where the climate of Odessa is contrasted with that of the S.W. shores of the Chersonesus, sheltered by mountains, and fit for the cultivation of the olive and the orange-tree.

2dly. Along the Gulf of Genoa, from Toulon and the Hieres Isles to Nice and Bordighera, (*Annales du Museum*, tom. xi. p. 219,) where the small maritime palm-tree, *chamærops*, grows wild, and where the date-tree is cultivated on a large scale, not to obtain its fruit, but the palms or etiolated leaves.

3dly. In England, on the coast of Devonshire, where the port of Salcombe has, on account of its temperate climate, been called the Montpellier of the North, and where (in South Hams) the myrtle, the *camellia Japonica*, the *fuchsia coccinea*, and the *buddleia globosa*,* pass the winter in the open ground, and without shelter.

4thly. In France, on the western coasts of Normandy and Brittany. In the department of Finisterre, the arbutus, the pomegranate-tree, the *yucca gloriosa* and *aliofolia*, the *erica Mediterranea*, the *hortensia*, the *fuchsia*, the *dahlia*, resist in open ground the inclemency of a winter which lasts scarcely fifteen or twenty days, and which succeeds to a summer by no means warm. During this short winter, the thermometer sometimes falls to 17·6. The sap ascends in the trees from the month of February; but it often freezes even in the middle of May. The *lavatera arborea* is found wild in the isle of Glenans; and opposite to this island, on the continent, the *astragalus bajonensis* and the *laurus nobilis*.†

From observations made in Britanny for twelve years, at St. Malo, at Nantes, and at Brest, the mean temperature of the peninsula appears to be above 56·3. In the interior of France, where the land is not

---

* KNIGHT, *Trans. Hort. Soc.* vol. i. p. 32.—In 1774, an agave flowered at Salcombe, after having lived twenty-eight years without being covered in winter. On the coast of England, the winters are so mild, that orange-trees are seen on espaliers, which are sheltered, as at Rome, only by means of a matting.

† BONNEMAISON, *Geogr. Botan. du Depart. du Finisterre*, (*Journal de Botan.* tom. iii. p. 118.)

much elevated above the sea, we must descend 3° of latitude in order to find an annual temperature like this.

It is known, from the researches of Arthur Young,* that, in spite of the great rise of the two isothermal lines of 53·6 and 55·4 on the western coast of France, the lines of culture (those of the olive, and of the maize and vine,) have a direction† quite opposite, from S.W. to N.E. This phenomenon has been ascribed,‡ with reason, to the low temperature of the summers along the coast; but no attempt has been made to reduce to numerical expressions the ratios between the seasons in the interior and on the coast. In order to do this, I have chosen eight places, some of which lie under the same geographic parallels, and others in the prolongation of the same isothermal line. I have compared the temperatures of winter, of summer, and of the warmest months; for a summer of uniform heat excites less the force of vegetation, than a great heat preceded by a cold season. The terms of comparison have been along the Atlantic; the coasts of Brittany, from St. Malo and St. Brieux to Vannes and Nantes; the sands of Olonne; the Isle of Oleron; the embouchure of the Garonne and Dax, in the department of the Landes: and, in the interior, corresponding to the same parallel, Chalons sur Marne, Paris, Chartres, Troyes, Poitiers, and Montauban.

| Places in the Interior. | Latitude. | Mean Temperature | | | |
|---|---|---|---|---|---|
| | | *of the Year.* | *of Winter.* | *of Summer.* | *of the Warmest Month.* |
| | | Fahr. | Fahr. | Fahr. | Fahr. |
| Chalons sur Marne | 48·57 | 50·5 | 36·1 | 66·6 | 67·5 |
| Paris | 48·50 | 51·1 | 38·7 | 65·3 | 67·5 |
| Chartres | 48·26 | 50·7 | 37·0 | 64·6 | 65·7 |
| Troyes | 48·18 | 52·2 | 38·3 | 67·3 | 68·4 |
| Chinon | 47·26 | 53·4 | 38·7 | 69·1 | 70·2 |
| Poitiers | 46·39 | 54·3 | 39·7 | 67·1 | 69·3 |
| Vienne | 45·31 | 55·0 | 38·7 | 71·6 | 73·4 |
| Montanban | 44·01 | 55·6 | 42·6 | 69·3 | 71·4 |
| **Places on the Coast.** | | | | | |
| St. Malo | 48·39 | 55·5 | 42·4 | 66·9 | 67·5 |
| St. Brieux | 48·31 | 52·3 | 41·7 | 64·4 | 67·1 |
| Vannes | 47·39 | 51·8 | 39·7 | 64·4 | 65·8 |
| Nantes | 47·13 | 54·7 | 40·5 | 68·5 | 70·5 |
| La Rochelle | 46·14 | 53·1 | 40·3 | 66·6 | 67·1 |
| Oleron | 45·56 | 58·1 | 44·6 | 68·5 | 72·1 |
| Bourdeaux | 44·50 | 56·5 | 42·1 | 70·9 | 71·4 |
| Dax | 43·52 | 54·1 | 44·4 | 67 3 | 68·9 |

* *Travels in France*, vol. ii. p. 91.

† The line which limits the cultivation of the vine, extends from the embouchure of the Loire and of the Vilaine, by Pontoise, to the confluence of the Rhine and the Moselle. The line of the olive-trees commences to the west of Narbonne, passes between Orange and Montelimart, and carries itself to the N.E. in the direction of the Great St. Bernard.

Farther south, from $44\frac{1}{2}°$ of lat., the comparisons become incorrect, because France, locked between the Ocean and the Mediterranean, presents, along its last basin, in the fine region of the olives, a system of climate of a particular kind, and very different from that of the western coast.

These results are deduced from 127,000 observations, made with sixteen thermometers, of, no doubt, unequal accuracy. In supposing, on the theory of probabilities, that, in such a number of observations, the errors, in the construction and exposure of the instruments, and in the hours of observation, will in a great measure destroy one another, we may determine, by interpolation, either under the same parallel or upon the same isothermal line, the mean winters and summers of the interior and of the coast of France. This comparison gives—

| | | | Mean Winter. | Mean Summer. | |
|---|---|---|---|---|---|
| I. Isothermal Lines of | 52° 7 | Coast | 40 6 | 65·1 | |
| | | Interior | 38·5 | 68·0 | |
| | 54°·7 | Coast | 41·4 | 67·3 | |
| | | Interior | 39·2 | 68·4 | |
| | | | | | *Annual Temp.* |
| I. Parallels of | 47° to 49° | Coast | 41·0 | 66·7 | 53·0 |
| | | Interior | 37·8 | 66·6 | 51·6 |
| | 45° to 46° | Coast | 42·3 | 67·8 | 55·8 |
| | | Interior | 39·2 | 69·3 | 54·7 |

As the isothermal lines rise again towards the western coasts of France,—that is to say, as the mean temperature of the year becomes there greater than under the same latitude in the interior of the country,—we ought to expect that, in advancing from east to west under the same parallel, the heat of the summers would not diminish. But the rising again of the isothermal lines, and the proximity of the sea, tend equally to increase the mildness of the winters; and each of these two causes acts in an opposite manner upon the summers. If the division of the heat between these seasons was equal in Brittany and in Orleannois, in the climate of the coast and the continental climates, we ought to find the winters and summers warmer in the same latitude along the coast. In following the same isothermal lines, we readily observe, in the preceding table, that the winters are colder in the interior of the country, and the summers more temperate upon the coasts. These observations confirm, in general, the popular opinion respecting the climate of coasts; but, in recollecting the cultivation and the development of vegetation on the coasts and in the interior of France, we should expect differences of temperature still more considerable. It is surprising that these differences between the winter and the summer should not exceed 1·8, or nearly a quarter of the difference between the mean temperature of the winters or the summers of Montpellier and Paris. In speaking of the limits of the cultivation of plants upon mountains, I shall explain the true cause of this apparent contradiction. In the mean time it may be sufficient to remark, that our meteorological instruments do not indicate the quantity of heat which, in a clear and dry state of the air, the direct light produces in the more or less coloured parenchyma of the leaves and fruits. In the same mean temperature of the atmosphere, the development of vegetation is retarded or accelerated according as the sky is foggy or serene, and according as the surface of the earth receives only a diffuse light during entire weeks, or is struck by the direct rays of the sun. On the state of the atmosphere, and the degree of the extinction of light, depend, in a great measure, those phenomena of vegetable life, the contrasts of which surprise us in islands, in the interior of continents, in plains, and on the summit of mountains. If we neglect these photometrical considerations, and do not appreciate the production of heat in the interior of bodies, and the effect of nocturnal radiation in a clear or a cloudy sky, we shall have some difficulty in discovering, from the numerical ratios of the observed summer and winter temperatures of Paris and London, the causes of the striking difference which appears in France and England in the culture of the vine, the peach, and other fruit-trees.*

When we study the organic life of plants and animals, we must examine all the stimuli or external agents which modify their vital actions. The ratios of the mean temperatures of the months are not sufficient to characterize the climate. Its influence combines the simultaneous action of all physical causes; and it depends on heat, humidity, light, the electrical tension of vapours, and the variable pressure of the atmosphere. It is the last cause which, on the tops of mountains, modifies the perspiration of plants, and even increases the exhaling organs. In making known the empirical laws of the distribution of heat over the globe, as deducible from the thermometrical variations of the air, we are far from considering these laws as the only ones necessary to resolve all the problems of climate. Most of the phenomena of nature present two distinct parts,—one which may be subjected to exact calculation, and another which cannot be reached but through the medium of induction and analogy.

---

* Young's *Travels in France*, vol. ii. p. 195.

